# Corporate political activity of baby food companies in Thailand

**DOI:** 10.1186/s13006-021-00437-6

**Published:** 2021-12-23

**Authors:** Nisachol Cetthakrikul, Phillip Baker, Cathy Banwell, Matthew Kelly, Julie Smith

**Affiliations:** 1 Research School of Population Health, Australian National University, ACT, Canberra, Australia; 2 International Health Policy Program, Ministry of Public Health, Nonthaburi, Thailand; 3 Institute for Physical Activity and Nutrition, Deakin University, Geelong, Victoria Australia

**Keywords:** Corporate political activity, Baby food company, Policy process

## Abstract

**Background:**

Recent studies show corporate political activity (CPA) can have detrimental impacts on health policy processes. The Control of Marketing Promotion of Infant and Young Child Food Act B.E. 2560 (the Act) was implemented in Thailand in 2017, but there have been no studies documenting CPA during its policy processes. Furthermore, the effects of CPA on the Act and how non-industry stakeholders dealt with the CPA have not been explored. This study aimed to analyze the CPA of baby food companies in Thailand, its effects on the Act, and how policymakers have responded to CPA around the Act.

**Methods:**

This qualitative study applied an established framework developed by Mialon and colleagues to collect and systematically analyze publicly available information from seven baby food companies with the highest percentage market share in Thailand. In-depth interviews were also used to explore how people involved in the policy process of the Act experienced the CPA of baby food companies, the consequent effects on the Act, and how they responded to the CPA.

**Results:**

During development of the Act, baby food companies used two main strategies, ‘information and messaging’ and ‘constituency building’. We found the companies met policymakers, and they employed evidence or provided information that was favorable to companies. Also, they established relationships with policymakers, health organizations, communities and media. The effects of CPA were that the scope of products controlled by the Act was reduced, and CPA led relevant people to misunderstand and have concerns about the Act. Officials and others countered the influence of CPA by raising awareness and building understanding among involved people, as well as avoiding contact with companies informally.

**Conclusions:**

CPA consists of a variety of practices that resulted in a weakened Act in Thailand. Government officials and other non-industry stakeholders employed strategies to counteract this influence. This study suggests the Department of Health, and other relevant government agencies, would benefit from establishing safeguards and protections against CPA. Efforts to raise awareness about the harms of CPA within and outside of government and establish a systematic monitoring system, including avoid conflict of interest in policy process would improve policymaking and implementation of the Act.

**Supplementary Information:**

The online version contains supplementary material available at 10.1186/s13006-021-00437-6.

## Background

To achieve optimal growth, development and health the World Health Organization (WHO) recommends infants and young children are exclusively breastfed for the first 6 months of life, and thereafter receive nutritionally adequate and safe complementary foods, while breastfeeding continues for up to 2 years of age or beyond. The WHO/UNICEF *Global Strategy for Infant and Young Child Feeding* calls on governments to protect, promote and support breastfeeding through policy and programming action, including the adoption of *The International Code of Marketing of Breast-Milk Substitutes* and subsequent World Health Assembly resolutions (*The Code*) into national law. Adopted by the World Health Assembly in 1981, *The Code* is a response to long-standing concerns that the marketing of commercial breastmilk substitutes (BMS) undermines breastfeeding and harms infant, child and maternal health in all countries, irrespective of their development status [1].

In 1981, Thailand adopted The Code into national policy as a voluntary measure to control baby food marketing in the country. However, acknowledging the many violations of The Code both in Thailand [2, 3], and other countries [4–7], the National Health Assembly of Thailand passed a resolution that The Code should be strengthened to be a regulatory measure in 2010. In 2014, the Department of Health (DOH) and the Ministry of Public Health (MOPH), commenced the development of a draft Bill on food for infants and young children, adopted finally in 2017 as the Control of Marketing Promotion of Infant and Young Child Food Act B.E. 2560 (“The Act”) [8].

The action plan for implementation of the Act states that: “*the key objective of the Act is that baby food marketing is controlled appropriately and complies with international standards to protect rights and health of infants and young children in order to be fed appropriate food through protection mothers and families by receiving correct information about infant and young-child food”* [9]. The Act controls baby food promotion to mothers and the public through, for example, advertisements, providing information, discounts, free samples, free products, gifts, and direct contact with mothers. Furthermore, baby food marketing to health professionals through sponsorship and providing scientific information is also controlled by the Act. Likewise, the Act controls baby food marketing in health facilities, for example, through donation of products, and offering materials or equipment.

Corporate political activity (CPA) is the term used to describe companies’ attempts to shape government policy in ways that are favorable to the companies [10]. In other words, CPA refers to a set of practices employed by industries to influence government, policymakers, and the public, to interrupt, delay or terminate policy that affects them negatively. Industries have employed CPA to interfere with policy processes in order to limit policy development in their sector. A recent study in Thailand showed how the food industry in Thailand applied a variety of strategies and practices of CPA to influence government policy and public opinion, in ways that undermined efforts to prevent obesity and diet-related non-communicable disease (NCDs) [11]. As well as constituency building, information provision and messaging were key strategies observed [11]. A study of CPA by the dairy industry in France also found ‘information and messaging’ and ‘constituency building’, but also ‘policy substitution’ strategies, which involve the industry proposing alternatives such as voluntary initiatives or self-regulation when threatened by regulation [12].

Similar activities have been observed in the global baby food industry. For example, in the Philippines, the Department of Health provided Implementing Rules and Regulations (IRR) to restrict the marketing of baby food in 2006. The Pharmaceutical and Health Care Association of the Philippines, which at the time included baby food companies as members, sued the government of the Philippines arguing that the Department of Health exceeded their powers in enacting the IRR. They aimed to declare the IRR void by attempting to transfer the IRR legislature from the Committee on Health to the Committee on Trade in the House of Representatives. In addition, the United States Chamber of Commerce sent a letter to the President of the Philippines to state that the IRR had a negative effect on business. As a result, the IRR was restrained temporarily.

Although the Supreme Court rejected nearly all of the baby food industry’s claims in 2007, implementation of the IRR was delayed by 398 days due to industry interference [13]. Tanrikulu et.al studied the CPA of Nestlé in the United States of America in 2020 to identify and monitor the CPA of the baby food industry. Findings showed that Nestlé applied many CPA strategies which were ‘instrumental strategies’, ‘information strategy’, ‘establish relationships with non-industry stakeholders, ‘seek involvement in community’, ‘direct involvement and influence in policy’ and ‘discursive strategies’. The CPA may have been influential in shaping policies in ways favorable to the baby food industry [14].

In 2015, Mialon et al. provided a CPA framework in relation to the food industry and public health [15]. The framework shows six strategies of CPA namely: information and messaging; financial incentives; constituency building; legal; policy substitution; opposition fragmentation and destabilisation, along with practices and mechanisms for each strategy (please see additional file 1). Many studies employ this CPA framework to analyze CPA, for example in the food industries in France [16], Australia [17, 18], Fiji [19], Chile [20] and Thailand [11].

To date, no studies have examined the CPA of baby food companies during the policy process [21] of the Act in Thailand. Therefore, this study aimed to describe and analyze the CPA of baby food companies in Thailand to understand whether their activities negatively affected the process of policy development. In addition, this study sought to understand how relevant people such as government officials, academics and non-government organizations (NGOs) who were involved with policy process of the Act, dealt with CPA, so lessons can be learned for further regulatory activities.

## Methods

This study adopted an exploratory qualitative design using interviews with key informants. To understand the CPA of the baby food industry during the policy process, we were guided by i) a ‘stages of the policy process’ theory acknowledging that policy proceeds through agenda setting, formulation, adoption and implementation stages; and ii) the framework developed to identify and categorize the CPA of the food industry developed by Mialon et al. [15]. The approach proceeded through three steps: identification of baby food companies, data collection, and data analysis.

### Selection of samples

#### Baby food companies

The seven baby food companies with the highest percentage of market share in Thailand [22] were selected for inclusion in the study. The selected companies were Nestlé (Thai) Ltd., Dumex Thailand Co Ltd. (Danone), Mead Johnson Nutrition (Thailand) Ltd., FrieslandCampina (Thailand) PCL, Abbott Laboratories (Thailand) Ltd., Heinz Thailand Ltd., and Peachy Village Co Ltd. In addition, this study included Pacific Healthcare (Thailand) Co Ltd. because it is a transnational company, producing goat’s milk marketed for infants and young children who have an allergy to cow’s milk. Therefore, the total number of baby food companies in the study was eight (see Table 1).

**Table 1:** Company shares of baby food

Companies’ name	Market share (%)
Nestlé (Thai) Ltd	40
Dumex Thailand Co Ltd. (Danone)	34.1
Mead Johnson Nutrition (Thailand) Ltd	16
FrieslandCampina (Thailand) PCL	4.5
Abbott Laboratories (Thailand) Ltd	2.2
Other	3.2

### Data source and data collection

#### Key informants

Key informant interviews provide valuable knowledge and perspective on a specific topic. Thirty-four key informants who were involved in the policy process (agenda setting, policy formulation, policy adoption and policy implementation) of the Act were selected through purposive sampling and snowball sampling techniques. Firstly, we asked a government official who was responsible for maternal and child health at the Department of Health (DOH) to list key informants involved with policy processes of the Act. Secondly, the key informants were contacted for interviews. Lastly, the key informants were asked for names and contact information of other relevant people involved in policy processes.

The key informants comprised 28 government officials; two university professor academics; and four leaders of non-governmental organizations (Table 2).

**Table 2:** Number of key informants

Type of key informants	Number
Government official, Central level	6
Government official, Regional level	6
Government official, Provincial level	16
Academic	2
NGOs	4
**Total**	**34**

#### Systematic approach to collecting publicly available information

This study collected CPA materials produced by baby food companies from publicly available documents on the Internet. A web search was applied to collect data from companies’ sources namely: 1) company websites (Thai website only) and social media (Facebook, Instagram); and 2) website of the Pediatric Nutrition Manufacture Association (PNMA). Additionally, data were collected from online sources of government organizations that may conducted activities with baby food companies: websites of universities with a school/department of nutrition/dietetics/maternal and child health; the website of Parliament and Senate, and the websites of departments or agencies responsible for health. Matichon Online, the largest newspaper database in Thailand, was another source of data.

Publicly available documents relating to CPA published between 1 January 2010 and 31 December 2019 were included in this study to explore CPA during the policy process of the Act. The data collection was conducted from a web search conducted between January and April 2020. Also, the name of baby food companies and their products’ name were applied as search terms.

#### In-depth interviews

The interviews were conducted from April to July 2020. The key informants were interviewed via telephone or online channels using the Line application and Zoom, which was due to the coronavirus pandemic. Interviews lasted between 30 to 90 min for each key informant. The key informants were asked about firstly, CPA undertaken by baby food companies during the policy process of the Act, and secondly, their reaction to the CPA. The last question concerned the effects of CPA on the Act. During in-depth interviews, an audio recorder was used for sound recording, and a researcher took notes.

### Data analysis

All documents relating to CPA were retrieved from relevant websites. Then, the documents were read rigorously to group them according to the framework [15]. Likewise, sound recordings from interviews were transcribed verbatim, and then we used ATLAS.ti 8 to organize the transcriptions. Using inductive and deductive thematic analysis [23], we read and coded the transcriptions to identify CPA strategies, practices, and mechanisms.

Codes from documents and quotations from interviews, presented in this article, were translated from Thai to English by a researcher, and they were reviewed and edited by fluent Thai-English speakers, to ensure the meaning was not changed. Furthermore, codes were represented by the letter “A” in this article (please see additional file 2).

## Results

### The CPA of baby food companies during the policy process

There were 447 publicly available documents relating to the CPA of baby food companies in Thailand between 2010 and 2019; 271 documents presented ‘information and messaging’ strategies, which was the highest number of retrieved documents, and the number of the documents showing ‘constituency building’ strategies was 176 (Table 3).

**Table 3:** The number of publicly available documents relating to CPA of baby food companies and number of key informants who mentioned CPA strategies

Strategies	Practices	Number of documents (*n* = 447)	Number of key informants (*n* = 34)
1 Information and messaging	1.1 Lobby policymakers	0	6
	1.2 Stress the economic importance of the industry	9	0
	1.3 Promote deregulation	0	0
	1.4 Frame the debate on diet- and public health-related issues	60	0
	1.5 Shape the evidence base on diet- and public health-related issues	202	2
2 Financial incentive	2.1 Fund and provide financial incentives to political parties and policymakers	0	0
3 Constituency building	3.1 Establish relationships with key opinion leaders and health organizations	58	12
	3.2 Seek involvement in the community	103	7
	3.3 Establish relationships with policymakers	0	7
	3.4 Establish relationships with the media	15	0
4 Legal	4.1 Use legal action (or the threat thereof) against public policies or opponents	0	0
	4.2 Influence the development of trade and investment agreements	0	0
5 Policy substitution	5.1 Develop and promote alternatives to policies	0	0
6 Opposition fragmentation and destabilization	6.1 Criticize public health advocates	0	0
	6.2 Create multiple voices against public health measures	0	0
	6.3 Infiltrate, monitor and distract public health advocates, groups and organizations	0	0
Total		**447**	

*CPA* Corporate political activity

Most key informants talked about relationship establishment with key opinion leaders or health organizations (*n* = 12), and seven of them mentioned seeking involvement in community or establishing relationships with policymakers (Table 3).

### Information and messaging

Strategies relating to information and messaging can be found from both publicly available documents and interviews. Baby food companies applied four practices namely to ‘lobby policymakers’, ‘stress the economic importance of the industry’, ‘frame the debate on diet- and public health-related issues’, and ‘shape the evidence base on diet and public health-related issues’.

#### Lobbying policymakers

The publicly available documents did not illustrate lobbying. However, key informants said that baby food companies met or sent letters to policymakers and relevant people who were involved with the policy process of the Act, directly or indirectly. The examples of people who met with baby food companies were chief executive officers of the Ministry of Public Health (Minister, Permanent Secretary, Director General and Deputy Director-General of the Department of Health), committees and members of the National Legislative Assembly, Officers of the National Economic and Social Development Council, and columnists of newspapers.

*When we were drafting the Act, companies would ask to meet with the Director of [the] Department of Health, Permanent Secretary and Minister. . . (Government official)*

*The Chair of committee of National Legislative Assembly told me that the company sector had approached him already, but they hadn’t discuss [ed] the matter in detail . . . (NGOs)*

Baby food companies talked to relevant people to argue or present their concerns over the Act.

*I remembered exactly, a baby food company showed up with a media representative. They informed us that they had issued a letter to request a meeting with the Minister in order to provide information, along with the media company . . . When the Minister walked in, they approached him and said, “Today, I came with [baby food company’s representative name] to discuss my concerns over the Act.” Then, the representative of the baby food Association expressed the [ir] concerns toward the Act . . . (Government official)*

#### Stressing the economic importance of the industry

Publicly available documents revealed that companies stressed the economic importance of the industry by emphasizing local workers’ employment; for example, companies addressed the number of Thai workers who were hired by the companies (A1 − A2). Furthermore, the documents showed that baby food companies provided knowledge to Thai farmers such as how to increase the quality of cow’s milk, or farm management, by training or study visits. Companies said that the farmer had more income or profit as a result (A3 − A7). However, no key informants mentioned anything relating to this practice.

#### Frame the debate on diet- and public health-related issues

The practice of “fram [ing] the debate on diet and public health-related issues” was evident in publicly available documents only. Baby food companies promoted their good intentions to support breastfeeding (A8 − A9) and nutrition (A10 − A14). To show the positive traits of the food industry, the baby food companies also stressed that their products were good quality and nutritious (A15 − A17), including that they followed the criteria of international organizations (A17). They also claimed to promote and improve the health of children (A18 − A20), and the general population (A21). Furthermore, companies said they support the health-related goals of international organizations (A20 − A21) and that they had social responsibility (A22).

Companies emphasized the food industry’s actions to address public health-related issues. Firstly, baby food companies said they did research to improve their products (A23 − A24). Next, the companies emphasized that they hosted activities or events involved with health and nutrition with activities targeting children (A25), mothers (A19, A26 − A27), and the general population (A28 − A31).

#### Shaping the evidence-base on diet and public health-related issues

This study also identifies the practice of “Shap [ing] the evidence based on diet and public health-related issues”. Baby food companies funded academic research (A32 − A36), and some baby food companies have their own research institute for product development (A18, A37 − A42). In addition, findings illustrated there were some experts who claimed that nutrients, child development and child health were linked to the products of baby food companies (A43 − A46).

Baby food companies selected data that favored the companies by making a connection between information or results of studies and companies’ products (A40, A47 − A51). For example, companies mentioned health problems such as malnourishment, vitamin deficiency or allergies, and presented that their products had complete and sufficient nutrients, or that their products contained 100% partially digested whey protein in order to reduce molecule size, which can mitigate the risk of children having allergies.

Another practice used by baby food companies was participation in and hosting of scientific events. The companies hosted or participated in both offline (A49, A52 − A85) and online events (A86 − A135) to provide information or to convey messages to target groups. Physical events or activities were targeted at parents/care-givers (A52 − A72), health professionals (A73 − A77) and the media (A49, A78 − A81), while the target group of all virtual events was parents/care-givers. Furthermore, the publicly available evidence revealed that some physical scientific events were collaborative meetings between the baby food companies and private hospitals (A53 − A54, A65 − A66) or universities (A67, A84) or health professional associations (A72, A75).

Key informants reported that baby food companies used a third person who was credible in the media such as newspapers to represent their concerns over the Act. In other words, companies avoided publicly expressing their concerns themselves but used others to exercise influence on issues affecting their commercial interests.

Around the time when the Act was about to be adopted, they [companies] employed some people to publish concerns in the newspaper that the Act . . . Those people who were expressing concerns were not from companies, so I think it’s their tactic to avoid expressing their concerns over the Act. (Government official)

### Constituency building

#### Establishing relationships with key opinion leaders and health organizations

Findings from the analysis of documents show that baby food companies established relationships with key opinion leaders and health organizations by promoting public-private interactions. For instance, the companies cooperated with health professionals to develop a mobile application for mothers providing guidelines on child-raising(A136).

Similarly, baby food companies promoted public-private relationships with the university sector by supporting universities’ activities such as meetings conducted by a university (A137), field trips which were a part of study subjects (A138), and university projects (A139 − A142). Furthermore, published documents showed that the baby food companies had cooperative arrangements or partnerships with government organizations (A143 − A149). For example, companies collaborated with government organizations to promote a project to enhance dairy farm management for Thai dairy farmers.

In addition, publicly available evidence showed that baby food companies provided materials, equipment or money to public hospitals (A150 − A151).

In terms of establishing informal relationships with key opinion leaders, publicly available documents revealed that baby food companies provided companies’ publications to key opinion leaders (A152). Key informants reported the companies sought to contact health professionals in health facilities.

*. . . prohibited them [companies] to contact pregnant women, but it was evident that they still contact the pregnant staff anyway. (Government official)*

*The baby food company was not prohibited from approaching doctors in hospitals, right? So, they [companies] contacted pediatricians to conduct with them Video conferences. (Government official)*

Moreover, key informants observed that baby food companies had built relationships with health professionals for a long time, for example through sponsoring travel.

*Simply put, it had been a long-term built relationship. In the past, doctors and nurses were invited by companies to travel, and they agreed to join the trip. (Government official)*

#### Seeking involvement in the community

Public documents presented that baby food companies sought involvement in the community by donating their products to victims of natural disasters such as floods (A153 − A160), or they gave the companies’ products to poor children at foster homes (A161 − A171, A183), including providing food to children (A170). The companies also provided their products to schools in communities (A172).

Apart from the donation of products, baby food companies donated money to government organizations including universities and foster homes to help poor children (A173 − A176). In addition, they gave items to government organizations to distribute to mothers, such as publications produced by the companies (A177). Also, the companies had volunteer activities in communities (A178). This included building libraries for communities.

Baby food companies also supported events at community level. Most commonly, companies hosted events or activities focusing on health (A172, A181 − A192), and supported events for youth (A193 − A195) which was similarly reported in interview data from key informants. The companies also supported swimming activities for mothers with infants (A179 − A180).

Key informants observed that baby food companies made contact with local organizations such as Sub-district (or Tambon) Health Promoting Hospitals, Sub-district Administrative Organizations and Provincial Administrative Organizations in order to conduct events or activities in districts or sub-districts.

*[Companies] approached a local leader to participate in 3 Aor 2 Sor [local Health promotion program] . . . but they were also promoting their stage 3 formula [growing-up milk] by showing products off during the event. (Government official)*

Key informants mentioned that after the Act was implemented, baby food companies preferred to enter communities through local administration organizations since they did not want to violate the Act.

*… Sometimes if they [companies] realized that [health] officers . . . had been trained, they [companies] wouldn’t host an event or violate the regulation in hospitals. However, they [companies] would conduct activities to provide knowledge at the local community instead. Though they [companies] would not mention milk products, they would use words such as supplementary food or healthy food instead. (Government official)*

#### Establishing relationships with policymakers

The practice of “establishing relationships with policymakers” was not evident in publicly available documents, but interviews with key informants showed that baby food companies established relationships with policymakers by seeking involvement in working groups, technical groups and advisory groups. Particularly, in the process of policy formulation and adoption, baby food companies attempted to attend the meeting of the committee of National Legislative Assembly for consideration and review drafts of the Act.

*When it [the Act] was just a draft, I was also [on] one of the committees of National Legislative Assembly. At that time, I noticed how they [companies] were trying to disguise themselves into the committees as well. (Government official)*

However, after the Act was adopted, companies wanted to be on committees of the Act, or working group.

*They [companies] tried to infiltrate the committee [of the Act] or a working group on behalf of the association of baby food companies . . . (NGOs)*

Furthermore, the companies provided technical support and advice to relevant people or organizations between policy formulation and policy enforcement by giving information or suggestions to adjust or revise the Act, which was done through committees of the Act or formal letters.

*When the Act was still a draft, . . . they [companies] sent letters to the Prime Minister, Parliament, and Minister of Public Health to express their opinion that they disagreed with some wordings in some chapters, and they wanted to adjust them into their preferred words. (Government official)*

Also, the baby food companies established informal relationships with policymakers by meeting new executives.

*Mostly, when executive people change, they [companies] will approach the new executives. (Government official)*

#### Establishing relationships with the media

Documents revealed that baby food companies conducted media meetings to provide information relating to maternal and child nutrition or health (A49, A78 − A81, A137). They also introduced their new products to the media, for example though an editor of a newspaper (A196), including, inviting representatives of the media on field trips to visit their research institutes (A197). Additionally, the companies established informal relationships with influencers or celebrities, for example, the companies posted congratulatory messages for a newborn baby of an actress (A198). Nevertheless, key informants did not mention this practice.

### Impacts of CPA on Thailand’s the Control of Marketing Promotion of Infant and Young ChildFood Act B.E. 2560

Key informants said that CPA of baby food companies had many effects on policy processes of the Act. First and foremost, the content of the law was weakened.

*It is obvious that they [companies] can successfully weaken the Act. The main point was that [the Act] doesn’t cover stage 3 formula [growing-up milk] . . . We [Department of Health and network] proposed that [the Act] should cover up to stage 3 formula, right? . . . However, eventually, these tactics [CPA] had weakened [the Act] which only included up to one year old [milk formula] . . . (Academic)*

The Act was weakened because CPA applied pressure from the public.

*We [Department of Health] were pressured by academics, maternal and child experts, as well as from politicians to amend and lessen [the Act] enforcement . . . Thus, from a perspective of a person who had a strong sense toward these matters, this [the Act] might seem comparatively weak when considering how it was first drafted. (Government official)*

However, some other key informants reported that the Act was revised or amended due to legislation processes, and the Act as first drafted by the Department of Health needlessly repeated others previous regulations. Also, the authorities did not have sufficient evidence to support specific wording in the Act.

*The first draft [of the Act] from the Ministry [of Public Health] was fine . . . But when it [draft of the Act] was edited by committees of the Council of State, it [content of the Act] was deducted. One possible reason is that the Act cannot be written with such specific detail and it [content of the Act] had to be removed and replaced with legal wording instead . . . Admittedly, it [content of the Act] did not cover all expected details as first launched from the Department of Health [first draft of the Act] . . . (Government official)*

CPA by baby food companies also created misunderstandings among the public or other relevant people. Consequently, they became concerned about the Act, or developed a negative attitude to breastfeeding or the Department of Health. The result was that enforcement of the Act was affected.

*I went to [government organization] [to] explain [about the Act] because [government organization] sent a letter that showed their [government organization] concern. They [government organization] said that a representative of the company had already approached them [government organization]. (Government official)*

On the other hand, some key informants thought that CPA did not have any impact on law implementation and law enforcement because it depended on the processes of the Department of Health.

*In terms of advocating [the Act], we [Department of Health] can perform our work following the timeline and our processes without interruption. In term [s] of content, as I said, it was up to the vast majority of the committees [of the Act]. When we [Department of Health] launched guidelines or recommendations, it [guideline or suggestion] would follow the vast majority of the committees’ opinion [of the Act]. (Government official)*

### The public responses to CPA of baby food companies

Key informants revealed that they developed ways of coping with CPA of baby food companies. Firstly, they gained awareness of relevant people, especially, health professionals like ‘miss nom-mae’ (a lactation consultant) and became more wary of involvement with CPA, for example through companies establishing relationships with key opinion leaders and health organizations.

*I embedded the awareness among [lactation consultants] to not only promote breastfeeding, but also [lactation consultants] to protect [breastfeeding] . . . So . . . they [lactation consultants] confirmed that [if there is any contact with companies] they will refuse immediately . . . they [lactation consultants] weren’t interested in any incentive. (Government official)*

Secondly, the DOH built an understanding about the Act with relevant organizations and policymakers during policy formulation and the policy adoption period to clarify or explain about the Act, since companies had met or contacted relevant organizations, and policymakers.

*If we [DOH] know [that companies have contacted the official department] and we [DOH] are able to fix it on time such as [government organization], we will be able to revert the situation because [government organization] was one of organizations that the cabinet needs in order to get their [government organization] approval on the Act. At first, we sent [draft of the Act] to them [government organization] and they [government organization] disagreed with it, but when we approached them [government organization], they [government organization] then agreed with it. (Government official)*

The DOH had a technical team, chaired by the Deputy Director-General of DOH. The team prepared information and documents during policy formulation and policy adoption, in order to explain about the draft Act to the interested public and legislators.

*… The Department of Health had gathered [letters or questions from companies or other non-industry stakeholders] beforehand, and then [Department of Health] analyzed their [companies’ or* non-industry *stakeholders’] opinions and reactions, in order to plan our [Department of Health] course of action. [I] think that it was a team of the Deputy Director-General [of Department of Health] . . . At that time, the draft [of the Act] was still under the consideration of National Legislative Assembly where they [Department of Health] still had to explain [about the Act to National Legislative Assembly] and some documents were still withheld by National Legislative Assembly. And there were questions periodically about the Ministry’s opinion and editions [on the draft of the Act] to conform with opinions from the Royal College, milk companies, and Associations or Government officials.*

After implementation of the Act, there is a working group for which the Department of Health played a role as the Secretariat and sought information to support policy implementation and enforcement.

*There is a working group, with the Department of Health as a secretariat . . . when there were issues, we [working group] will find information to provide support [issues] . . . (Government official)*

There are committees, sub-committees and working groups of the Act. Baby food companies wanted to be a part of committees, sub-committees or working groups, but it was not appropriate. Therefore, the committees of the Act denied this to the companies.

*They [companies] tried to be a part of committees or working groups . . . but the committee considered it inappropriate to allow industrial stakeholders to be involved in policy processes or creating law enforcement’s details . . . It would be as if we allowed alcoholic drinks or tobacco companies to be part of policy making process which is ironic. They tried to offer their [companies] hands but we deemed it inappropriate . . . (NGOs)*

After the law’s implementation, companies sought involvement in the community instead of establishing relationships with key opinion leaders and health organizations. Locals did not understand or know about the Act. To build understanding with locals and to avoid being involved with CPA, responsible people communicated about the Act with locals.

*At the local level, they [locals] didn’t know. If someone gives them something or provides support, they considered it as a good thing. But this time, I conducted conferences in my district in a meeting-like manner . . . and then I invited locals in order to explain [about the Act] . . . Sometimes, they [locals] didn’t understand, but [I] tried to explain to them, because joining [meeting] only once, they might [locals] not understand . . . as it [the Act] is hard to understand . . . (Government official)*

Authorities and relevant people, who in relation to legislation, avoided informal contact with companies, still heard companies’ and other non-industry opinions.

*At first, they [companies] phoned us to raise issues where [I] didn’t interrupt because it was rude . . . Mostly, [I] told [companies] to send a letter in order to mitigate conflict . . . (Government official)*

## Discussion

This study is the first to investigate the CPA of baby food companies in Thailand and their influence on legislation regulating inappropriate baby food marketing. We applied a systematic approach using the analytical framework of the CPA of the food industry for public health [15], to collect and analyse available documents and to conduct in-depth interviews, with the aim to collect data on CPA of baby food companies existing between 2010 and 2019. We also examined the impact of CPA on the development of the Control of Marketing Promotion of Infant and Young Child Food Act B.E. 2560, and how relevant people responded to the CPA by baby food companies.

Both document review and in-depth interviews found that two strategies were used: ‘information and messaging’ and ‘constituency building’. Our results showed that the baby food companies in Thailand used eight out of sixteen practices identified in the CPA framework [15]. There were four practices from the ‘information and message’ strategy: lobbying, stressing the importance of the industry to the economy, promoting the good intentions of companies, and shaping research evidence relating to diet and public health. Likewise, they used practices relating to ‘constituency building’ through establishing relationships with four relevant groups: policymakers, communities, health professionals and the media.

This is a similar finding to a previous study in the USA, which showed that Nestlé, as an example of the baby food industry, employed an information strategy by funding, producing and disseminating industry-favorable information [14]. Also, Nestlé established relationships with key opinion leaders, health organizations and the media, and sought involvement in communities. Moreover, the company used indirect access and the placement of representatives in governments to influence policies and programs. The company also applied discursive strategies to argue over diet- and public health-related issues.

There were three practices that can be found from both resources, namely ‘Shaping the evidence base on diet- and public health-related issues’, ‘relationship establishment with key opinion leaders and health organizations’ and ‘seeking involvement in the community’. ‘Stressing the economic importance of the industry’, ‘framing the debate on diet- and public health-related issues’ and ‘establishment of relationship with media’ were evident in publicly available documents. Meanwhile, lobbying and relationship establishment with policymakers was addressed by the key informants only. This is because in Thailand, there is no register of lobbyists, unlike Australia [24] or Canada [25]. Consequently, there were no publicly available documents relating to lobbying.

According to publicly available documents, shaping the evidence base on diet and public health-related issues was found in 200 practice examples and seeking involvement in the community was related to one-third (*n* = 103) of practice examples. In in-depth interviews, the establishment of relationships with key opinion leaders and health organizations was mentioned by 12 key informants, while seven key informants mentioned either establishing a relationship with policymakers or seeking involvement in community.

Our study is also related to the findings of studies of the global marketing activities of the baby food industry. In 2017 Granheim et al. found that baby food companies applied six tactics used by the tobacco industry to interfere with public health policy. The tactics were maneuvering to hijack the political and legislative process; exaggerating the economic importance of the industry; manipulating public opinion to gain an appearance of respectability; fabricating support through front groups; discrediting proven science; and intimidating governments with litigation, to interfere public health policy [13]. These tactics can all be found in this study with the exception of intimidating governments with threat of litigation.

Hastings et al. found that baby food companies sought to build relationships with mothers and health professionals [26]. The companies tried to connect with mothers through Baby Clubs and providing telephone advice and digital technology. Companies also linked with health professionals by providing financial and educational support in order to establish relationships. In the same way, this study found that baby food companies established relationships with health professionals and key opinion leaders by building educational and financial links with them and they conducted activities for mothers in order to establish relationships with them.

In 2020 Baker found that baby food companies have the power to influence others directly by hiring lobbyists to engage policy decision-makers or employing law firms for litigation [27]. As well as engaging health professionals and scientists, they establish front groups and civil society groups. The companies became more important for national economies due to their market growth. Also, they had power to influence the production of knowledge and evidence. Likewise, the results of this study illustrate that baby food companies tried to meet policymakers during the formulation of the Act. They employed a third person to express their concerns about the Act, such as limiting consumer access to information. Furthermore, the findings showed that companies tried to stress the economic importance of the industry. They can shape evidence because they have their own research institutes and hosted scientific conferences or forums to provide cherry-picked information to target groups.

The policy process is potentially subject to industry influence at various stages, as shown in the theoretical and empirical literature. For example, in Australia the implementation of a public education resource, designed to support a new government food labelling initiative, was delayed due to an inappropriate relationship between policymakers and food manufacturers [28]. Likewise, a recent study in Fiji [19], showed strong relationships between the food industry and the Ministry of Trade which had negative impacts on the development and implementation of public health measures. A policy substitution strategy stopped the Ministry of Health from developing or implementing new NCDs regulations [19].

In Chile [20], food companies attended technical meetings conducted by government, which led to delayed decision-making. A study of the CPA of the Australian food industry illustrated that by establishing relationships with policymakers, CPA had negative impacts on the development of effective public health policies [17]. The last example is a study of CPA of the food industry in Thailand, with results revealing that lobbying can interrupt government initiatives for prevention of obesity and diet-related NCDs [11]. Similarly, this study found that baby food companies have built relationships with health professionals for a long time, and they tried to meet policymakers to express their concerns on the Act.

This study showed that the Act and the content of the Act was likely to have been influenced by the CPA of baby food companies acting in their commercial interests. The narrowing in the scope of products significantly weakened the Act by removing the growing-up milk formula category. Also, CPA led relevant people who were involved with the policy processes of the Act to misunderstand, become concerned about or form negative attitudes about the Act, such as relating to providing information about the baby food aspect of the Act. Consequently, the contents of the Act are different from the Bill which the Department of Health proposed in 2015. Moreover, a study about trade policy of World Trade Organization (WTO) and infant and young child nutrition policies found that WTO processes had negative impacts on the Act. This is because these processes allowed BMS importers from United States of America, New Zealand, Australia, the European Union, and Canada to express their concerns about the Act that may restrict goods trade or discriminate against imports, and compliance of the Act with Codex standards through the WTO Technical Barriers to Trade committee. Finally, the Act was weakened by allowing the advertisement of growing-up milk [29] (See additional file 3).

Industry reports on the baby food market in Thailand in 2018 record that growth in milk formula sales are constrained by the new regulation, and the industry has redirected its marketing activity [22]. The growing-up milk formula became the first priority for advertisement because it is not prohibited to be advertised as it is not the same as other categories of baby food which are not permitted to be advertised. Also, baby food companies made an effort to increase brand awareness as a whole, not just focused on a particular product. Moreover, they hosted activities or event for mothers to increase their engagement with their brand [22]. This is consistent with findings in Australia since 1992, where companies have opposed the inclusion of follow-up milk in regulatory responses to the 1981 WHO Code resolution, and then began heavily marketing such products [30].

In response to CPA, authorities and relevant people gained awareness and built understandings about CPA specifically targeted at people working in policy processes of the Act such as policymakers, the Cabinet, and the committee of the Office of the Council of State etc. Also, code watchers and community leaders gained awareness and built understandings. Another response to CPA was that government officials working on legislative processes avoided informal contact with baby food companies; however, they heard opinions from all sectors, particularly, baby food companies.

In 2016, WHO launched the ‘Guidance on ending the inappropriate promotion of foods for infants and young children: implementation manual’ [31]. The guidance recommended that companies must take responsibility to avoid any marketing which creates conflicts of interest. Health workers, health systems, health professional associations and non-governmental organizations should also avoid conflicts of interest.

This study points to the need to equip policymakers and regulators with strategies and tools to effectively manage CPA influence on policy and regulatory decisions during the development of new legislation or regulations.

This study had some limitations. Firstly, key informants were asked to recall the CPA of baby food companies that happened between 2010 and 2019 and may not have been able to remember the CPA exactly or some important details of CPA may not been mentioned. A second limitation was that some CPA was unlikely to be recorded, or records were contained in confidential documents of the companies; this study retrieved only publicly available documents. The result of this was that not all CPA was collected in our data. Last but not least, most selected companies (seven out of eight companies) in this study were multinational companies due to their percentage of market share, so the CPA of local or smaller companies was not included. Whether local or smaller companies’ CPA is different in strategies and impact could be an important area for future research.

Future research needs to explore which CPA are the most effective at interrupting policy processes in order to identify priority prevention measures.

### Policy implications

The 2003 Global Strategy identifies the obligation of baby food companies to abide by the Code and comply with Codex Alimentarius standards. While the Code does not use the term Conflict of Interest (COI), several subsequent WHA Resolutions address this issue [32]. The 2016 Resolution has recommendations for member states, manufacturers, health care professionals, media and creative industries, and civil society on avoiding conflicts of interest, and specifically calls on manufacturers not to create conflicts of interest.

Major international food companies have used several strategies from the international ‘playbook’ to exert influence on Thailand’s legislation on inappropriate baby food marketing, acting in their commercial interest, and against the public interest. Some CPA prevention was not covered by the Act, for example, providing technical support and advice to policymakers, or funding research. However, other CPA of baby food companies still aimed to affect and did affect the policy development process of the Act. Thai authorities, and society members employed many strategies to cope with the CPA targeted at them, which provides lessons for future regulation.

To strengthen the Act implementation and enforcement, the Department of Health and Government could protect the Act from baby food companies’ interference in policy through a revision that prohibits the baby food companies from participating in policy processes. A good model for strong action is provided in Article 5.3 of the Framework Convention on Tobacco Control [33]. For example, it recommends specific measures for raising awareness about industry policy interference, avoiding conflicts of interest for government officials and employees, and requiring information from industry including on its marketing expenditures and lobbying activities. It also recommends denormalising and regulating corporate social responsibility activities such as those identified in Thailand on baby food. As well, there needs to be more transparency about CPA. For instance, any interactions of the government or civil society with industry or its allies should be limited and made immediately transparent.

Furthermore, recent research has identified baby food industry activities to influence Codex Alimentarius standards, including in ways which interfered with Thailand implementing the Code fully to include toddler formulas [29]. This highlights the importance of exposing industry CPA at international level and specifically on the urgent need to remove industry influence from CODEX standard setting processes.

## Conclusion

Based on the findings of this study, the policy recommendations are as follows. First, the Department of Health must have systems and measures in place to deal with CPA of baby food companies, such as staff training, and a communication strategy to provide information relating to CPA which may lead them to have a conflict of interest, as well as how to respond CPA. This will ensure that CPA will not weaken the Act’s implementation, enforcement and other processes. Second, relevant people such as knowledgeable and competent officers, health professionals and committees should be trained to fully understand the CPA of companies in order to recognize and deal with the issue, so they can avoid involving the companies in key stages of developing policy and regulation or legislative measures. Lastly, the Department of Health could have monitoring systems to monitor the CPA of baby food companies regularly to detect new practices or strategies of CPA. Consequently, the Department of Health can better prepare itself to cope with the CPA.

## Supplementary Information

**Additional file 1.** Framework of CPA of food industry with respect to public health [1].

**Additional file 2.** Codes relating to CPA from publicly available documents.

**Additional file 3.** Key different issues between bill and the Control of Marketing Promotion of Infant and Young Child Food Act B.E. 2560.

## Data Availability

The data that support the findings of this study are available from online sources such as companies’ websites and their social media, universities’ websites, website of Parliament and Senate, websites of departments or agencies responsible for health and Matichon e-library, but some sources like Matichon e-library have restrictions which apply to the availability of these data because data can be accessed by members only.

## Abbreviations

BMS: Breastmilk substitutes
CPA: Corporate political activity
DOH: Department of Health
MOPH: Ministry of Public Health
NCDs: Non-communicable diseases
NGOs: Non-government organizations
USA: United State of America
WHO: World Health Organization
WTO: World Trade Organization
