# Supplementary material for: Corporate political activity of baby food companies in Thailand
Source: Int Breastfeed J. 2021 Dec 23;16:94. doi: 10.1186/s13006-021-00437-6 (PMC8696240; doi:10.1186/s13006-021-00437-6)
Supplement: Supplementary file 2 — Additional file 2. Codes relating to CPA from publicly available documents. [file 13006_2021_437_MOESM2_ESM.pdf]

Additional file 2: Codes relating to CPA from publicly available documents

ID	Source	Data coded	Strategy	Practice (code used for analysis)
A1	Company website	With over 45 years in Thailand, [company's name] currently employs more than 500 people………..	Information and messaging	Stress the economic importance of the industry
A2	Company social media	[Company's name] encouraged new generation to join working market. In 2016, 352 new graduates were recruited…	Information and messaging	Stress the economic importance of the industry
A3	Company social media	[Company's name] support Thai dairy farmers by knowledge dessimination in order to increase cow's milk quality, and create income route among Thai dairy farmer sustainably …	Information and messaging	Stress the economic importance of the industry
A4	Company website	Not only that [Company's name] was one of the main buyers who bought more than 100 million litres of raw cow milk from Thai dairy cooperative, we also support Thai dairy farmers to improve skills of farm management...	Information and messaging	Stress the economic importance of the industry
A5	Company social media	Nowadays, we provide knowledge for Asian dairy farmers through training session and sponsoring site visits in the Netherlands. [Name of company's program] has already assisted and supported more than 40,000 dairy farmers across Asia by helping farmers improving the quality of cow's milk. As a result,some dairy farmers can earn up to 25% higher income.	Information and messaging	Stress the economic importance of the industry
A6	Company social media	[Company's name] intended to help farmers who raised cows by focusing on improving quality and quantity of milk in order to raise profits by providing suggestion on hygenic maintainance of milking set and a bucket as well as steps of cow milking accordingly to hygienic principle in order to build consumers' trust on milk's quality.	Information and messaging	Stress the economic importance of the industry
A7	Company social media	[Company's name] provided knowledge and equipped dairy farmers with a skill to plant Napier grass, an easy-to-grow forage and high yield per field. Nowadays, more than 90% of Dairy Cooperative members operated their own Napier grass fields which can reduce the expenditure needed for green forage. They can also obtain milk with higher percentage of milk fat which can raise a better selling price for milk.	Information and messaging	Stress the economic importance of the industry
A8	Company social media	[Name of company's club] support and promote "breastfeeding"	Information and messaging	Frame the debate on diet- and public health-related issues
A9	Company social media	[Company's name] encourage everyone to support "breastfeeding"	Information and messaging	Frame the debate on diet- and public health-related issues

ID	Source	Data coded	Strategy	Practice (code used for analysis)
A10	Company website	At [Company's name], the positive attitude, perseverance, pride and passion in working to help provide the best start of life for children are traditions that have been carried in every employee’s heart and these qualities are passed along to every [Company's name] employee in Thailand	Information and messaging	Frame the debate on diet- and public health-related issues
A11	Company website	[Company's name] strives to present food and beverage products with good quality and nutrition standard.	Information and messaging	Frame the debate on diet- and public health-related issues
A12	Company social media	[Company's name] strives to produce nutritious food with good taste to support 50 million children around the world to be healthy.	Information and messaging	Frame the debate on diet- and public health-related issues
A13	Matichon	... continues to improve the quality of life for people at all life course by using scientific-based nutrition and health ...	Information and messaging	Frame the debate on diet- and public health-related issues
A14	Company website	[Company's name] strives to be recognized as a leader of the world on nutrition to become healthy and have good quality of life.	Information and messaging	Frame the debate on diet- and public health-related issues
A15	Company social media	Every carton of [product's name] has a quality control process using Focus system... every cartons was produced punctiliously and thoughtfully from the beginning until finished…because we want everyone to drink a quality milk…	Information and messaging	Frame the debate on diet- and public health-related issues
A16	Company website	The vision of [company's name] is to provide good nutrition to customers; and has been committed to that for more than 60 years. We, thus, constantly develop high quality products in order to ensure nutrients from 100% high quality cow's milk for customers.	Information and messaging	Frame the debate on diet- and public health-related issues
A17	Company website	[Company's name] is a leader in producing modified milk for children. We share the same acknowledgement with other health organizations around the world that breast milk is the best food for a baby. However, if the baby cannot be breastfed, the modified milk product of [company's name] provides high nutrients, and our products meet certification standards, and comply with regulations of countries around the world including the International Code of Marketing of Breastmilk Substitutes - or WHO Code.	Information and messaging	Frame the debate on diet- and public health-related issues

ID	Source	Data coded	Strategy	Practice (code used for analysis)
A18	Matichon	...The main mission ... is to encourage every child to have a good start for the highest ability in life ... We have researched and found that children who are well-raised from the earliest stage of life will be healthy and show a quality growth ... These have led to two strategies… 1. Innovation ... the company has established [Name of research institute] ... by linking technology and research ... as well as providing new knowledge to academics and health professionals ... 2. Social responsibility with a determination to provide a proper nutrition as a good start for children ...	Information and messaging	Frame the debate on diet- and public health-related issues<br><br>Shape the evidence base on diet and public health-related issues
A19	Company social media	The global goal of [company's name] is to help 50 million children around the world to have a better quality of life for a better health by 2030 which there is a project [project name] that gathers activities executed by [company's name] …in order to support parents and care givers to raise a child healthily.	Information and messaging	Frame the debate on diet- and public health-related issues
A20	Company social media	[company's name] strives to improve quality of life of more than 50 million children to be healthier by 2030, and jointly drive sustainable development goals of United Nations…	Information and messaging	Frame the debate on diet- and public health-related issues
A21	Company social media	[company's name] has three important aims to improve population health and environmental conservation for our world as well as a determination to support United Nations sustainable development goals	Information and messaging	Frame the debate on diet- and public health-related issues
A22	Company website	[Company's name] has operated programs that acted as a guideline for implementing the social and environmental policies across all business groups of [Company's name] around the world. Each company can assess their performance by choosing assessment criteria from more than 100 criteria which cover social responsibilities such as food safety, human resources, environment and relationship with partners and customers. Therefore, we merged economic and social environment in planning work in each country that [company's name] group has run a business.	Information and messaging	Frame the debate on diet- and public health-related issues

ID	Source	Data coded	Strategy	Practice (code used for analysis)
A23	Matichon	... [product name] assimilates a specialist in maternal and child nutrition that has earned trusts from Thai families for more than 80 years. We have never stopped performing researches and develops for better suitable innovative products for mothers during pregnancy and lactation including nutritional supplement products for children aged 1 year old or beyond ...	Information and messaging	Frame the debate on diet- and public health-related issues
A24	Company website	In each year [company's name] gives priority to researches and developments with the budget over 80,000 million Baht per year in order to increase quality of life and health promotion toward the future.	Information and messaging	Frame the debate on diet- and public health-related issues
A25	Company social media	[Name of the company] promote a better life for Thai Children through the campaign [Name of the campaign] which aims to distribute knowledge among Thai youth about healthy food consumption and living a healthy life.	Information and messaging	Frame the debate on diet- and public health-related issues
A26	Matichon	...Organize a project to enhance knowledge towards sustainability of nutritional education in order to develop new way of nutritional training to educate parents at child care centers across the country ...	Information and messaging	Frame the debate on diet- and public health-related issues
A27	Matichon	…Launched a new campaign...to encourage modern mothers to breastfeed their child for a longer period of time…	Information and messaging	Frame the debate on diet- and public health-related issues
A28	Matichon	...conducting [event name]... to encourage Thai people to drink 100% fresh cow milk regularly for at least 2-3 cups per day to bring about a 100% healthy Thai families.	Information and messaging	Frame the debate on diet- and public health-related issues
A29	Matichon	...[Company's name] launched a new campaign in 2018 [campaign name]…this campaign emphasized on [company's name]'s determination to increase quality of life and promote good health toward the future.	Information and messaging	Frame the debate on diet- and public health-related issues
A30	Matichon	...The company has proceeded with a large campaign ... under [project name] and ready to move forward to expand the product groups that have been certified with 'Healtier choice' logo from the Ministry of Public Health ... and increased to 50 items by this year ...	Information and messaging	Frame the debate on diet- and public health-related issues
A31	Matichon	[Company's name] paid 40 million THB to elevate nutrition and healthy standard…we are spending budget on improving communication and conducting activities to promote nutrition and health balancing for all Thai people…	Information and messaging	Frame the debate on diet- and public health-related issues

ID	Source	Data coded	Strategy	Practice (code used for analysis)
A32	university website	[Company's name] gave 8,281,234	Information and messaging	Shape the evidence base on diet and public health-related issues
A33	University website	[Study’s name] with [expert's name] as principal investigator. The study's budget was from [company's name]	Information and messaging	Shape the evidence base on diet and public health-related issues
A34	University website	Research fund [name of research fund]	Information and messaging	Shape the evidence base on diet and public health-related issues
A35	University website	Research fund from company [company's name]	Information and messaging	Shape the evidence base on diet and public health-related issues
A36	Matichon	…the research project [research project name] supporting by of the company under study of [university name] ...	Information and messaging	Shape the evidence base on diet and public health-related issues
A37	Matichon	...Behind this success was the continuous research to create innovations and new products to be launched in market, the main research center [research institute name] ...as an innovative research institute ...	Information and messaging	Shape the evidence base on diet and public health-related issues
A38	Matichon	...[research institute name]...It's the largest and most advanced food and nutrition research center in the world ...[company's name] has allocated a budget of more than 60 billion Baht devoted to research and developing products for health ...	Information and messaging	Shape the evidence base on diet and public health-related issues
A39	Matichon	... To strengthen and to stimulate child's immune system after breast milk, [research institute name] which is a well-known nutritional research institute among European countries, has performed research on substances or ingredients of food that will help strengthen immune system in children...	Information and messaging	Shape the evidence base on diet and public health-related issues
A40	Matichon	... According to the findings from [research institute name], a global expert in early life nutrition, research was conducted among sample of healthy children between the age of 1-3 years ... which [research institute name] has followed up the physical changes of more than 767 children, who were in child care centers... in five countries in Europe and Asia, including Thailand, for 1 year	Information and messaging	Shape the evidence base on diet and public health-related issues
A41	Matichon	... Studies of [research institute name] has investigated various dimensions of nutritional impacts at early stage of life, …nutrients and components of breast milk, which has led to many innovations ...	Information and messaging	Shape the evidence base on diet and public health-related issues

ID	Source	Data coded	Strategy	Practice (code used for analysis)
A42	Matichon	[company's name] has opened [research institute name] …this center will be a utilized for product development for Asian people ...	Information and messaging	Shape the evidence base on diet and public health-related issues
A43	Matichon	[company's name] cooperated with a nutritionist to provide tips for food selection and introduce important nutrients, essential amino acids, and omega 369 which can contribute to effective baby's brain development ...[expert name] pointed out that ... Nutrients that can help with baby's brain development were essential amino acids and omega ...	Information and messaging	Shape the evidence base on diet and public health-related issues
A44	Matichon	Researchers addressed that Alpha-Lactalbumin contributed to children's brain development … [expert name] stated that...adding Alpha-Lactalbumin into milk powder can play an important role in better brain's functions.	Information and messaging	Shape the evidence base on diet and public health-related issues
A45	Company website	[Expert name] ... illustrated that obesity prevention can be performed as early as during infancy by breastfeeding at least 6 months and continuing for as long as possible... but if mothers are unable to breastfeed due to any conditions, choosing high-quality protein milk with proper amount that are relatively close to breast milk will help stimulating the body's metabolic system and reduce the risk of obesity as well as other severe chronic diseases. In addition, 100% partially digested whey protein can contribute to a reduced risk of allergic ...	Information and messaging	Shape the evidence base on diet and public health-related issues
A46	Matichon	With the care from [Company's name], the [Company's Name] then cooperated with pediatricians and nutritionists in order to provide recommendations about a double protection method for children during winter season [Name of expert]… there were several ways to strengthen immune system among children, but the best way is to strengthen natural immune system. Nowadays, innovation for strengthening immune systems has already been discovered namely Symbiotic…	Information and messaging	Shape the evidence base on diet and public health-related issues

ID	**Source**	**Data coded**	**Strategy**	**Practice (code used for analysis)**
A47	Company social media	In 2013, findings from research project and survey of [research name]...found that two thirds of Thai children were malnourished, with lower calcium, iron, vitamin D and vitamin A than recommended standard of WHO. Also, they showed slower development in comparison with other children in other countries. Nutritionist [research name] suggested that mothers immensely value nutrition by providing food or drink with complete and sufficient nutrients such as calcium, iron, vitamin D and vitamin A, in order to promote good health among babies as well as a proper development accordingly to their age.	Information and messaging	Shape the evidence base on diet and public health-related issues
A48	Matichon	[Product name] ...focuses on parents who are seeking for nutrients that are found in breast milk. ...The research findings certified that [nutrient name] will promote brain cell connections. Research also suggested that infants who consumed [nutrient name] containing milk showed similar brain and cognitive development to infant those who were fed with breast milk ...	Information and messaging	Shape the evidence base on diet and public health-related issues
A49	Company website	[company's name] conducted a seminar to provide knowledge among media…to suggest how to protect development of allergies among children since infancy age which provide a better long-lasting effectiveness.... Results of long-term studies in infants in Germany found that special milk [milk name] which contains 100% partially digested whey protein in order to reduce molecule sizes can mitigate the risk of having allergies among children in the long run ...if mothers cannot breastfeed, it is recommended to choose a special milk [milk name] that contains 100% partially digested whey protein which will reduce the risk of having allergies"[expert name] stated ...	Information and messaging<br><br>Constituency building	Shape the evidence base on diet and public health-related issues<br><br>Establish relationships with the media

ID	Source	Data coded	Strategy	Practice (code used for analysis)
A50	Company social media	"Recently, there was the first ever discovery from the study performed among children aged between two and 5 years old in the US. The results demonstrated that there was a correlation between an increased brain function and increased Myelin cells among children ...Hence, it can be concluded that Myelin is one of the most important factors that can contribute to children's brain development especially among young children whose brain develop rather rapidly. One of the essential nutrients for Myelin construction is Fatty acid namely Sphingo Myelin which can be found in breast milk, eggs, milk, cream cheese, etc.	Information and messaging	Shape the evidence base on diet and public health-related issues
A51	Matichon	...Recently, it was discovered that goat milk powder can provide a very high nutrient values .... This research has been performed among 301 Australian infants ... The results of the study confirmed nutritional benefits for infants in perspective of physical development, intelligence as well as infants immunity	Information and messaging	Shape the evidence base on diet and public health-related issues
A52	Matichon	[Product name] collaborated with leading experts in child's development to conduct a workshop "meeting experts and promote development in the right dimension"	Information and messaging	Shape the evidence base on diet and public health-related issues
A53	Company social media	[Hospital name] is inviting pregnant women and people who were interested in attending a lecture on the topic "A hundred billion brain cells, development toward the miracle of infinite learning. Sub-topic "preparing your baby's brain in your womb with a genious code"…	Information and messaging	Shape the evidence base on diet and public health-related issues
A54	Company social media	[Hospital name] and [product Name] would like to invite mothers to attend a meeting on the topic. "Modern mother cares about quality" by [expert name] on Sunday, August 30, 2015 at [hospital Name]…	Information and messaging	Shape the evidence base on diet and public health-related issues
A55	Matichon	... Organized an event "[activity name]" to encourage modern mothers to understand the importance of complementary food that are age-appropriate in each stages of children's development	Information and messaging	Shape the evidence base on diet and public health-related issues
A56	Matichon	... organized a seminar on the topic of ''baby is safe from having allergies, by starting at mothers and nutrition'' to address the importance of choosing the appropriate nutrition for baby to reduce the risk of having allergies ...	Information and messaging	Shape the evidence base on diet and public health-related issues
A57	Matichon	... organized a seminar to educate .... On the topic of L-Protextus, nutritious microorganism which can escalate toddler’s immunity ...	Information and messaging	Shape the evidence base on diet and public health-related issues

ID	Source	Data coded	Strategy	Practice (code used for analysis)
A58	Matichon	... organize seminars to provide knowledge about the importance of Bifidus BL on infant health and immunity development ...	Information and messaging	Shape the evidence base on diet and public health-related issues
A59	Matichon	... organized a forum "Investigate until can be certain, allergies are preventable" provided by the speaker [expert name] ...	Information and messaging	Shape the evidence base on diet and public health-related issues
A60	Matichon	...organized a seminar to disseminate "knowledge about nutrition for the development of pre-school children by [expert name] ...	Information and messaging	Shape the evidence base on diet and public health-related issues
A61	Matichon	...[Company's name] ... organized an academic seminar on the topic of "protecting babies from allergies, by starting with nutrition" in order to present the results of studies on nutrition for allergies prevention. ...This seminar was honored by world-class pediatrics experts ...	Information and messaging	Shape the evidence base on diet and public health-related issues
A62	Company website	[Product name] ... and [hospital Name] would like to invite mothers of children aged 1 year and over to participate in a workshop ... on Saturday October 1, 2016, at 10:00 AM - 12:30 PM ... there is a pediatrician who is an expert in child development and behavior to provide information about behavior of young children and the essential nutrients that parents should opt for their baby.	Information and messaging	Shape the evidence base on diet and public health-related issues
A63	Company website	...[Company's name] organized a training to provide nutrition knowledge for mothers of children aged 6 months to 3 years...which was supported by a team of nutritionists from [Name of University] In addition, [company's name] has also provided healthy snacks for children at [foundation name].	Information and messaging<br><br>Constituency building	Shape the evidence base on diet and public health-related issues<br><br>Seek involvement in the community
A64	Company website	[Company's name] organized activities [activity name] ... Providing knowledge and consultation on nutritional problems for pregnant women, women who are breastfeeding as well as recommendations for infant nutrition, by professors from [university name] ...	Information and messaging	Shape the evidence base on diet and public health-related issues
A65	Matichon	... [Company name] and [hospital name] ... provided knowledge on topics ''Confident in your baby's health in any seasons “, for modern parents to be ready for the weather changes, get ready to handle colds and diseases that come along with the rainy season ...	Information and messaging	Shape the evidence base on diet and public health-related issues
A66	Company social media	"[Hospital Name] and [Company's Name]" "Invited to join a panel discussion by medical professionals on" "Raising children to be smart in both IQ&EQ" "on August 20, 2011, 1:30 - 4:00 pm.	Information and messaging	Shape the evidence base on diet and public health-related issues

ID	Source	Data coded	Strategy	Practice (code used for analysis)
A67	Company social media	[Company club name] and [university name] would like to invite parents to experience and learn techniques to pave a better future for your beloved child in the event "[activity name]"...Exciting activities for pregnant women and join the forum leading by national experts ...	Information and messaging	Shape the evidence base on diet and public health-related issues
A68	Company social media	Invited parents to practice raising children by joining workshops with experts who have expertise at raising children at the event [activity name]…	Information and messaging	Shape the evidence base on diet and public health-related issues
A69	Matichon	... organized a brain exhibition ...and invited experts and celebrity families to join the event …	Information and messaging	Shape the evidence base on diet and public health-related issues
A70	Matichon	[expert name] ... was invited by [company's name] to provide knowledge on how to support child development ...	Information and messaging	Shape the evidence base on diet and public health-related issues
A71	Company website	[Company's name] ...organized an activity to educate on baby weaning stage. ... The event was honored by [expert name] ... to answer questions about "Yoghurt consumption for Kids" along with a tip sharing by celebrity mother [celebrity name] about how to raise a baby to be healthy, and a showcase in cooking yogurt menu ...	Information and messaging	Shape the evidence base on diet and public health-related issues
A72	Matichon	...a grand medical seminar [seminar name] ... at [company's name] ... cooperated between [company's name] and [medical association] ... [expert name] presented about nutritional innovation for prevention and treatment of allergic diseases ...	Information and messaging	Shape the evidence base on diet and public health-related issues
A73	Matichon	... [company's name] ...conducted a large medical seminar [Conference name] to gather medical professionals from various countries in Asia-Pacific countries. 500 world-class doctors from Europe also participated to share knowledge ...	Information and messaging	Shape the evidence base on diet and public health-related issues
A74	Matichon	[Company] conducted an academic seminar on the topic of Branergy Complex DHA Plus ... where doctors and health professionals also attended.	Information and messaging	Shape the evidence base on diet and public health-related issues
A75	Company social media	Nutrition for before born baby is more important than you have imagined… [company's name] and [medical association name] have hosted academic seminars for more than 300 doctors and nurses to emphasize an importance of nutrition for before born baby…	Information and messaging	Shape the evidence base on diet and public health-related issues
A76	Company website	[Company's name] organised the booth at [conference name]…	Information and messaging	Shape the evidence base on diet and public health-related issues

ID	Source	Data coded	Strategy	Practice (code used for analysis)
A77	Matichon	… [Company's name] ...conducted a medical seminar ...which there were more than 150 top class doctors in Thailand joined and exchanged ideas	Information and messaging	Shape the evidence base on diet and public health-related issues
A78	Matichon	... The company organized a seminar to educate media on the topic "A good future, starting with love from mom" and introduce the latest innovative products …this seminar was honored by [expert name]	Information and messaging<br><br>Constituency building	Shape the evidence base on diet and public health-related issues<br><br>Establish relationships with the media
A79	Matichon	...Recently [Product name] by [Company name]... organized a press conference on a topic "be knowledgeable on nutrition for preschoolers development" ...	Information and messaging<br><br>Constituency building	Shape the evidence base on diet and public health-related issues<br><br>Establish relationships with the media
A80	Company website	[Company's name] organized a press conference on the topic "The first step in motherhood is being thoughtful on good nutrition "… to address the importance of appropriate nutrition for mothers…	Information and messaging<br><br>Constituency building	Shape the evidence base on diet and public health-related issues<br><br>Establish relationships with the media
A81	Matichon	...seminar was conducted to educate the press on the topic "Build a good immunity today, and pave a way for a baby good health" in order to provide a reliable knowledge on benefits of Bifidus on immune system, digestive system and multi-dimensions of infant's development ...by [expert name]	Information and messaging<br><br>Constituency building	Shape the evidence base on diet and public health-related issues<br><br>Establish relationships with the media
A82	Matichon	...Organized an event [event name] at [conference name] ...	Information and messaging	Shape the evidence base on diet and public health-related issues
A83	Company social media	…"[activity name] build a new kid generation with high capacity in all dimensions ... "meet with pediatricians who are experts in child development…	Information and messaging	Shape the evidence base on diet and public health-related issues
A84	University website	Seminar on the topic "Nutrition seminar series" with [company's name]	Information and messaging	Shape the evidence base on diet and public health-related issues
A85	Matichon	...Academic seminar on the topic "Prevention is better than cure, allergies can be eliminated'' by [company's name] ... to present the results of a study on "An expenditure caused by allergies treatment among children from the age of newborns to 5 years old" by [expert name]	Information and messaging	Shape the evidence base on diet and public health-related issues
A86	Company social media	[online forum name] [expert name] on the topic "How to successful breastfeed and possible unknown nutritional knowledge for mothers"	Information and messaging	Shape the evidence base on diet and public health-related issues
A87	Company social media	Don't miss today at 1.30 pm. Join a discussion with expert doctor on the topic "How do mothers know that your lovely baby has allergies"...in [online forum name]	Information and messaging	Shape the evidence base on diet and public health-related issues

ID	Source	Data coded	Strategy	Practice (code used for analysis)
A88	Company social media	Show all the tips and places to take your child on vacation, invite mothers to talk to an expert today starting at 1.30 pm. in [online forum name]	Information and messaging	Shape the evidence base on diet and public health-related issues
A89	Company social media	Invite mothers to a live discussion on the topic ''ever since be pregnant, have you chosen the best things for your baby? " in [online forum name]	Information and messaging	Shape the evidence base on diet and public health-related issues
A90	Company social media	Invite mothers to a live discussion on the topic ''What kind of stomachache that mothers should not be ignoring" in [online forum name]	Information and messaging	Shape the evidence base on diet and public health-related issues
A91	Company social media	Don't miss live discussion tomorrow, on "how to make a first step of going to school a success one"	Information and messaging	Shape the evidence base on diet and public health-related issues
A92	Company social media	Live discussion about child's development with [expert name]… in [online forum name]	Information and messaging	Shape the evidence base on diet and public health-related issues
A93	Company social media	Live chat with expert [online forum name] "How can my child develop a healthy brain & immunity?"	Information and messaging	Shape the evidence base on diet and public health-related issues
A94	Company social media	...Live chat with experts [online forum name] on the topic "How to build immunity for your children when they are going to school" with [expert name]…	Information and messaging	Shape the evidence base on diet and public health-related issues
A95	Company social media	...Live chat with experts [online forum name] on the topic "Good nutrition for pregnant women and lactating mothers"…	Information and messaging	Shape the evidence base on diet and public health-related issues
A96	Company social media	Today… [online forum name] on the topic "How does caesarean section effect your baby's immunity?" join and discuss with a medical professional at 1:30 pm…	Information and messaging	Shape the evidence base on diet and public health-related issues
A97	Company social media	[online forum name] live talk with [expert name]…on the topic "Differences between Cesarean section and natural birth"	Information and messaging	Shape the evidence base on diet and public health-related issues
A98	Company social media	Don't miss! Live chat with experts [online forum name]. Today at 1:00 PM on the topic "What is nutrition for the newborn? and how does it affect health and capacity of your child in the future"	Information and messaging	Shape the evidence base on diet and public health-related issues
A99	Company social media	Don't forget, Today at 1.30 pm. [online forum name] come to join us in solving the problem with [expert name]… on the topic "Did you know! Children's allergy to cow's milk protein is preventable and treatable."	Information and messaging	Shape the evidence base on diet and public health-related issues
A100	Company social media	...[online forum name] on the topic 'How to prepare basic needs for your children to be successful'.	Information and messaging	Shape the evidence base on diet and public health-related issues

ID	Source	Data coded	Strategy	Practice (code used for analysis)
A101	Company social media	[Online forum name] on the topic 'Children can show different potential and skills. How to promote and bring out the best of their capacity?'' by [expert name]…	Information and messaging	Shape the evidence base on diet and public health-related issues
A102	Company social media	Pregnant ladies don't miss it !!!! Let's prepare a good health for your baby's future. ...[expert name] will answer all of concerning questions and provide advice on how to protect your baby from allergies in [online forum name]	Information and messaging	Shape the evidence base on diet and public health-related issues
A103	Company social media	[Online forum name] answers all concerned topics. 'Allergy to cow's milk…the beginning of any allergies that mother can be prepared for it. true or false? ''…	Information and messaging	Shape the evidence base on diet and public health-related issues
A104	Company social media	[Online forum name] invites all mothers to join a live chat on the topic "How to make your baby's first 1000 Days a healthy journey and how to encourage their highest potential" [expert Name].	Information and messaging	Shape the evidence base on diet and public health-related issues
A105	Company social media	Children are allergic to cow's milk, a big problem that mothers should not overlook. Come and join us to solve problems [expert Name]. ...in [online forum name]....	Information and messaging	Shape the evidence base on diet and public health-related issues
A106	Company social media	[Online forum Name] Mother, are you ready? Just log in to [link address] you will get to meet [expert Name], who will answer all concerns in the topic 'How to handle your child when they show unpleasant behaviours and how to recognize the signs of an unusual behaviour!!!	Information and messaging	Shape the evidence base on diet and public health-related issues
A107	Company social media	Today, don't miss !!!!! ...[online forum Name] meet again at 1:00 PM - 3:00 PM, ...[expert Name], will answer concerning questions about caesarean section…	Information and messaging	Shape the evidence base on diet and public health-related issues
A108	Company social media	...[online forum Name] Today at 1:30 PM - 3:30 PM. Meet [expert Name]…will answer concerning questions about " the developments in all pregnancy stages, and how to encourage a complete pregnancy "…	Information and messaging	Shape the evidence base on diet and public health-related issues
A109	Company social media	[online forum Name] on the topic of how to deal with the problem of cow's milk allergy by [expert name]…	Information and messaging	Shape the evidence base on diet and public health-related issues
A110	Company social media	... [online forum Name]…on the topic “How to observe and deal with the situation when your baby has a stomach discomfort symptom”…	Information and messaging	Shape the evidence base on diet and public health-related issues
A111	Company social media	... For all mothers who are especially concerning about breastfeeding, questions about nutrition on "How to eat safely and nutritiously during breastfeeding period, ...in [online Forum Name] with [expert Name] ...	Information and messaging	Shape the evidence base on diet and public health-related issues

ID	Source	Data coded	Strategy	Practice (code used for analysis)
A112	Company social media	...inviting all mothers, save up questions, come and consult with [expert name]… in [online Forum Name] ...	Information and messaging	Shape the evidence base on diet and public health-related issues
A113	Company social media	Facebook Live on the topic "Proactive parents guide a child toward a positive goal ” by [expert name]…and special guest [guest name] are ready to share a positive parenting experience…	Information and messaging	Shape the evidence base on diet and public health-related issues
A114	Company social media	Now, [expert name] is answering the questions through the program [online forum name]. Mothers who have a question about positive parenting or would like to know how to practice this method. Please send questions to the doctor. The doctor will be with us until 3:30 PM only!	Information and messaging	Shape the evidence base on diet and public health-related issues
A115	Company social media	[online forum name] on the topic… how to prepare an enjoyable and safe vacation …On August 25, 1:30 - 3:30 PM by [expert name]…	Information and messaging	Shape the evidence base on diet and public health-related issues
A116	Company social media	Prepare to meet the live broadcast [online forum name] at 1:30 PM. Unlock all the questions about allergies ''Preventing is better ...treatment is a big deal by [name of the expert], welcome mothers to watch and leave comments here…	Information and messaging	Shape the evidence base on diet and public health-related issues
A117	Company social media	...Meet with [expert name] who will come to answer to the concerned questions in [online forum name] on the topic "How does a Cesarean infant require a special care? ...	Information and messaging	Shape the evidence base on diet and public health-related issues
A118	Company social media	Come back again with [online forum name] in 3rd year, will be answering all the health problems of mothers and babies. This year we started with the topic. "How to take care of a baby with an allergy to cow's milk protein"	Information and messaging	Shape the evidence base on diet and public health-related issues
A119	Company social media	Meet the new style of [online forum name] via Facebook live, along with insights into the causes of allergies and how to prevent it by [expert name] ...	Information and messaging	Shape the evidence base on diet and public health-related issues
A120	Company social media	[Online forum name] on the topic "Nutrition in the First 1000 Days for a better Immunity"...	Information and messaging	Shape the evidence base on diet and public health-related issues
A121	Company social media	Started with a Live chat on the topic constipation and crying among your child can be prevented with alternative nutrition, anyone who have questions or concerns can leave questions under this comment ...	Information and messaging	Shape the evidence base on diet and public health-related issues

ID	Source	Data coded	Strategy	Practice (code used for analysis)
A122	Company social media	Wanting for children to have a discipline? find out how to raise children successfully to be disciplined and successful [expert name] ...anyone who have questions about the baby's behavior, leave questions under this comments…	Information and messaging	Shape the evidence base on diet and public health-related issues
A123	Company social media	Because a child's future success is attributed by a dedication of parents. Hence, to build a solid basics for your child to overcome all obstacles, encouraging obstacles' immunity is required. Today, [expert name] ... will educate and introduce techniques to build obstacles' immunity skill for children. Please send questions to consult in the comments box. Under this post.	Information and messaging	Shape the evidence base on diet and public health-related issues
A124	Company social media	[Online forum name] "Take your child to travel around the world, build immunity of obstacles for your child "...	Information and messaging	Shape the evidence base on diet and public health-related issues
A125	Company social media	[Online forum name] Answers all questions about "Cesarean Section", tips that mothers should know…	Information and messaging	Shape the evidence base on diet and public health-related issues
A126	Company social media	[Online forum name] ‘Allergy Gene can transmit From mother to children, if You know it beforehand you can prevent it from happening'... [expert name]…	Information and messaging	Shape the evidence base on diet and public health-related issues
A127	Company social media	[Online forum name] What should we choose "brain or immunity" for baby's nutrition in the first 1000 days?	Information and messaging	Shape the evidence base on diet and public health-related issues
A128	Company social media	Today, cow's milk protein allergy is a common disease, and the number has been increasing. Symptoms are various, so it is difficult to diagnose. If treatment is delayed, it may affect quality of the child's life in the long term. Do the parents know what is cow's milk protein allergy and what are the symptoms and how to take care of it? today [expert name]… will answer to the questions about cow's milk protein allergy.	Information and messaging	Shape the evidence base on diet and public health-related issues
A129	Company social media	Having a bedtime story, reading a book to your child will help with language development, imagination, and meditation, ...discover techniques for a proper selection of stories for each age group. ...by [expert name]...	Information and messaging	Shape the evidence base on diet and public health-related issues
A130	Company social media	Because the breast milk is the best for a baby. So what does it need to be done in order to maintain breastfeeding for the longest period of time? Come to see lactation stimulation techniques for new mothers to increase breast milk volumn by [expert name]... and [expert name]… Pregnant women and lactating mothers can send questions to ask the doctors in the comment…	Information and messaging	Shape the evidence base on diet and public health-related issues

ID	Source	Data coded	Strategy	Practice (code used for analysis)
A131	Company social media	In order to let children overcome obstacles ,both physically and mentally, and secure a succesful future... meet Facebook Live by [expert name]… ...ready to introduce techniques on how to immunize children to overcome obstacles…	Information and messaging	Shape the evidence base on diet and public health-related issues
A132	Company social media	[Online forum name]… “As a new parent, how to deal with child's allergy to cow's milk” by [expert name]… Any parents who have any questions about cow's milk allergy, prevention, treatment, leave a question in the comments.	Information and messaging	Shape the evidence base on diet and public health-related issues
A133	Company social media	...[Online forum name] on the topic "Uncomfortable stomach symptom in children. Solving it at its core can lead to uninterrupted development"...	Information and messaging	Shape the evidence base on diet and public health-related issues
A134	Company social media	[Online forum name] "What is a child undergrowth? Learn how to take care and solve problems "	Information and messaging	Shape the evidence base on diet and public health-related issues
A135	Company social media	[Online forum name] on the topic "Change allergy... to a ready to take all, to reduce allergies risks and look after a baby who has an allergy to cow's milk.	Information and messaging	Shape the evidence base on diet and public health-related issues
A136	Matichon	... we would like to suggest solutions on nutrtional topics developed by [name of the company's institution] ...and for development ... cooperates with the leading child development doctors and psychologists in Thailand for more than 1 year in order to develop the application ...to be guidelines to help mothers looking after their children	Constituency building	Establish relationships with key opinion leaders and health organizations
A137	University website	Providing knowledge about exercise to the press. Organized by [company's name]	Constituency building	Establish relationships with key opinion leaders and health organizations<br><br>Establish relationships with the media
A138	University website	Field trip of [subject name]…	Constituency building	Establish relationships with key opinion leaders and health organizations
A139	university website	[university name] and [company’s name] and network partners “[Project Name]…in an academic conference [conference name]…	Constituency building	Establish relationships with key opinion leaders and health organizations
A140	University website	[Company's name] and network partners operated [project name] ...to advocate the three health habits of Thai youths including eating a variety of foods, adding more fruits and vegetables, choose to drink more water, and increasing physical activities to pave a way toward a sustainable health among children aged 3-5 years by cooperating with all public and private organizations.	Constituency building	Establish relationships with key opinion leaders and health organizations

ID	Source	Data coded	Strategy	Practice (code used for analysis)
A141	Matichon	...Conduct a project [project name] of [company's name] and [university name] ...	Constituency building	Establish relationships with key opinion leaders and health organizations
A142	Company website	[Company name] collaborated with [university name] to run a new educational innovation project to develop feeding and nutrition guidelines for infants and young children in Thailand.	Constituency building	Establish relationships with key opinion leaders and health organizations
A143	Company social media	[Company's name] and [government organization] were cooperated to drive the "[project name]" to enhance dairy farm management for Thai dairy farmers for a better quality of life and sustainable career.	Constituency building	Establish relationships with key opinion leaders and health organizations
A144	Matichon	...The company supports government policies by participating [project name], sells at a lower price than the market price...	Constituency building	Establish relationships with key opinion leaders and health organizations
A145	Matichon	...[government organization] to honor the chairman of the event opening "[activity name]" ...	Constituency building	Establish relationships with key opinion leaders and health organizations
A146	Matichon	…The company collaborated with [foundation name] to continue activities to give back to the society …under the project [project name] ...	Constituency building	Establish relationships with key opinion leaders and health organizations
A147	Company social media	...[company's name] had a chance to perform activities together with [government organization]…	Constituency building	Establish relationships with key opinion leaders and health organizations
A148	Company social media	[Company's name] under the project "[project name]" was honored to participate in a lecture on the topic of "Nutrition for Early Childhood" at a seminar for developing early childhood practitioners' which organized by [government organization]. The goal is to enhance knowledge of nutrition and capacity building of Thai children …	Constituency building	Establish relationships with key opinion leaders and health organizations
A149	Company website	…[company's name]…support to conduct academic conference [conference name]…	Constituency building	Establish relationships with key opinion leaders and health organizations
A150	Company social media	…[company's name] provided a breast-milk corner which is under the project [project name] to [hospital name] ...	Constituency building	Establish relationships with key opinion leaders and health organizations
A151	Company website	...[company's name] donated money from sales and participated in project activities [project name] and donated to [hospital name] as a budget for necessary medical equipment …	Constituency building	Establish relationships with key opinion leaders and health organizations
A152	Company website	[company's name] attended the meeting and provided 100 books [book name] to [name of key opinion leader] ...at academic conference [conference name]…	Constituency building	Establish relationships with key opinion leaders and health organizations
A153	Matichon	...donated 550,000 units of milk ...along with organizing activities to reduce stress for people who immigrate to a shelter...	Constituency building	Seek involvement in the community

ID	Source	Data coded	Strategy	Practice (code used for analysis)
A154	Matichon	...donated 36,000 units of milk to ... [government organization] to deliver to the victims	Constituency building	Seek involvement in the community
A155	Company social media	[Company's name] ...continue to execute the project [Project name]... to donated 14,400 boxes of [product name] to help people with kind regard, in Ubon Ratchathani, who faced a heavy flooding …	Constituency building	Seek involvement in the community
A156	Company social media	...[Company's name] under the project [project name] provided [product name] valued at 120,000 baht through the flood victims rescue center at [university name] with [expert name] as the representative.	Constituency building	Seek involvement in the community
A157	Company social media	During the flooding incident [product name] donated [product name] to children who were evacuated to rescue centers…	Constituency building	Seek involvement in the community
A158	Company social media	The victims are not just only adults but there also babies and children who need food, as of now, by donating just 25 baht, you can send [product name]...to children...	Constituency building	Seek involvement in the community
A159	Company social media	[Product name] would like to be a part of helping all the Moken people at Mu Koh Surin…	Constituency building	Seek involvement in the community
A160	Company social media	...[product name] helped people in the south of Thailand who were flood victim ...	Constituency building	Seek involvement in the community
A161	Matichon	...Donated [product name] ...to children in foster home ...across the country...	Constituency building	Seek involvement in the community
A162	Matichon	...[product name] has continuously conduct a campaigned for drinking milk behavior by donating ready-to-drink milk to foundations and organizations ...	Constituency building	Seek involvement in the community
A163	Matichon	...Encourage you to send photos "Showing the world you are drinking milk " ...every photo sent will be exchanged into [Product name] to be given to [government organization] ...to distribute to underprivileged children…	Constituency building	Seek involvement in the community
A164	Matichon	...[product name] gave 100,000 boxes of milk …to many foundations to provide goods for underprivileged family	Constituency building	Seek involvement in the community
A165	Company social media	Let's give a smile, donated milk to the children in [foundation name] with [product name] on the World Milk Day…because [product name] wants everyone to support children to be healthy and have a proper development accordingly to their age…	Constituency building	Seek involvement in the community
A166	Company social media	…[product name] donated [product name] to the children at foster home [foster home name]…	Constituency building	Seek involvement in the community

ID	Source	Data coded	Strategy	Practice (code used for analysis)
A167	Company social media	…donated [product name] to children at [foster home name]…	Constituency building	Seek involvement in the community
A168	Matichon	...gave [product name] ...to ...[foster home name] ...	Constituency building	Seek involvement in the community
A169	Company social media	…[product name] has a chance to give [product name] to young girls at "[foster home name]"…	Constituency building	Seek involvement in the community
A170	Company website	[Company's name] ...donated [product name] and provide a meal to children at [foster home name]…	Constituency building	Seek involvement in the community
A171	Company social media	[Company's name] ...had a chance to visited children at [foster home name]…	Constituency building	Seek involvement in the community
A172	Matichon	...[product name] donated milks for a certain amount to the school ...educated the community about good nutrition that can be obtained from drinking milk ...for building up relationship with the community, [Product name] has also provided public health services to the community ...In addition, the company donated notebooks to schools in the community...	Constituency building	Seek involvement in the community
A173	Matichon	[Product name] donated 2.5 million baht ...as a scholarship to [government organization]...	Constituency building	Seek involvement in the community
A174	Matichon	...[Charity activity name] ...[product name] was one of the main supporters of this event ...all income ...have been dedicated for …food fund of [foster home name]	Constituency building	Seek involvement in the community
A175	Matichon	...company provides scholarships…to strengthen knowledge and abilities among students	Constituency building	Seek involvement in the community
A176	Company website	...[company name] donated money to help under privileged children in order to demonstrate gratitude toward society as well as to support the children at [foster home name]	Constituency building	Seek involvement in the community
A177	Company website	...[company name] gave 200 books to [government organization]…to hand over to child care center under [government organization]. The center can distribute knowledge among mothers continuously.	Constituency building	Seek involvement in the community
A178	Matichon	[company name] volunteered to help community to build library	Constituency building	Seek involvement in the community
A179	Company social media	Inviting to a seminar on the topic "Strengthen the development of babies and youth by swimming" On Saturday, September 27, 2014 at 9.00-12.00 and meet the booth of [product name]…	Constituency building	Seek involvement in the community

ID	Source	Data coded	Strategy	Practice (code used for analysis)
A180	Company social media	Let's swim:))	Constituency building	Seek involvement in the community
A181	Company website	Inviting mothers to attend [activity name], discover activities about children development and tips to raise children with care by heart…	Constituency building	Seek involvement in the community
A182	Company social media	[Company's name] and [supermarket name] organized the event "[activity name]". We recognized the importance and support in delivering good nutrition to all Thai children. ...Special !!! at [activity name] we have come to educate mothers about nutrition for their children by experts who are coming to answer about nutrition concerns...	Constituency building	Seek involvement in the community
A183	Company website	...[company's name] ...organized special activities [activity name] to give a chance to 3 winners ...join a three-week workshop to create a healthy lifestyle, both physically and mentally, under the supervision of 3 experts. ...The only one winner will get to make merit with [company's name] by delivering happiness via products …given by [company's name] ...to one of the three foundations chosen by the winner.	Constituency building	Seek involvement in the community
A184	Matichon	…the leading nutrition, health and wellness company takes its nutrition message to Thai most remote district…the project's activities aim to share comprehensive nutritional knowledge and promote physical and mental health through fun and educational learning	Constituency building	Seek involvement in the community
A185	Company website	[Company name] ...organized the event “[activity Name]” by inviting Thai consumers to join an enjoyable activity that inspire sustainable health…	Constituency building	Seek involvement in the community
A186	Company website	[company's name] ...conducted the biggest health campaign of the year [campaign name] On this occasion [company's name] invited consumers to join activities and receive special services, health check namely "age of the body" as well as receive "tips and weight management guidelines"...	Constituency building	Seek involvement in the community
A187	Company website	[Product name] by [company's name] is organizing an event [event name] ...there are various activities and knowledge to create solid background of success future by strengthening immunity…	Constituency building	Seek involvement in the community

ID	Source	Data coded	Strategy	Practice (code used for analysis)
A188	Matichon	...Organized [activity name] to provide happiness and good health to Thai people by participating in activities and receiving basic health checks with by an expert team…	Constituency building	Seek involvement in the community
A189	Company social media	[Company's name] supports Thai people to be sustainably healthy by organizing activities [activity name] a cross country to people in remote areas receive information about health care and nutrition.	Constituency building	Seek involvement in the community
A190	Company social media	...Inviting everyone to be healthy through good health and nutrition education activities, correct exercise and tips to manage your emotions to always be happy…	Constituency building	Seek involvement in the community
A191	Company social media	[Company's name] distributed healthy tips. One-by-one consultation with a nutritionist. Getting to learn about #food #exercise #emotion…	Constituency building	Seek involvement in the community
A192	Company website	[Company's name] ...conducted a press conference [press conference name] to announce direction of [company's name] to run business and social responsibility...we focus on creating value together with society in 3 main areas: development of nutrition for consumers. water resources management and community development ...[leader of community] said ...“[company name] recognized the needs of the community and have taken part in community development by providing knowledge of nutrition and health care for all members of the household ...	Constituency building	Seek involvement in the community
A193	Company social media	[Product name] want to spread happiness to the children at [foster home name] by using storytelling from their imagination…	Constituency building	Seek involvement in the community
A194	Matichon	...Organized a project at child care centers under the project name [Activity name] This activity are focusing on child development, care-giver training and environment adjustment to facilitate learning capacities for children.....	Constituency building	Seek involvement in the community
A195	Matichon	...Company cooperation with [foster home name] and [Local organization] recovered a learning center of preschool children under the project [project name]...	Constituency building	Seek involvement in the community
A196	Matichon	[company's name] brought products of company to introduce to editor of newspaper…….	Constituency building	Establish relationships with the media
A197	Matichon	...brought a group of Thai press ...to Switzerland in order to visit …[Research center name] ...	Constituency building	Establish relationships with the media

ID	**Source**	**Data coded**	**Strategy**	**Practice (code used for analysis)**
A198	Company social media	[company's name] congratulate to [name of influencers] in welcoming a little daughter…	Constituency building	Establish relationships with the media
