# Supplementary material for: Corporate political activity of baby food companies in Thailand
Source: Int Breastfeed J. 2021 Dec 23;16:94. doi: 10.1186/s13006-021-00437-6 (PMC8696240; doi:10.1186/s13006-021-00437-6)
Supplement: Supplementary file 3 — Additional file 3. Key different issues between bill and the Control of Marketing Promotion of Infant and Young Child Food Act B.E. 2560. [file 13006_2021_437_MOESM3_ESM.pdf]

Additional file 3: Key different issues between bill and the Control of Marketing Promotion of Infant and Young Child Food Act B.E. 2560

Issues	The Bill (August 2015)	Thai Milk Act (April 2017)
Growing-up milk	The Bill covered growing-up milk	Growing-up milk is included, but it has to be specifically proclaimed by the Minister with the advice of the Committee.
Incentive for health professionals	Do not give or offer any gifts, money, incentives, or other benefits to health professionals.	Companies can offer any gifts, money, incentives, or other benefits to health professionals if it is traditional giving.
Academic conference	Companies can provide funds to professional medical and public health organizations for academic conferences, training or seminar about baby food.	Companies can provide funds to professional medical and public health organizations; and public universities that offer curriculums on maternal and child health for academic conferences, training or seminar about baby food.
Information for health professionals	Companies can provide information to health professionals. The information must not provide incorrect or concealed or over-claimed or distorted information about baby food, also, the information dose not compare to breastfeeding.	In providing information about baby food to health professionals, the information should be supported by scientific evidence.
Labelling	Details of how to label, for example, labels of baby food products must have message “breastfeeding is the best”	The Act focuses on cross promotion by label only. There are no details about messages presented on labels.
